# Dengue Vaccine: Recommendations of the Advisory Committee on Immunization Practices, United States, 2021

**DOI:** 10.15585/mmwr.rr7006a1

**Published:** 2021-12-17

**Authors:** Gabriela Paz-Bailey, Laura Adams, Joshua M. Wong, Katherine A. Poehling, Wilbur H. Chen, Veronica McNally, Robert L. Atmar, Stephen H. Waterman

**Affiliations:** ^1^Division of Vector-Borne Diseases, National Center for Emerging and Zoonotic Infectious Diseases, CDC, San Juan, Puerto Rico; ^2^Wake Forest School of Medicine, Winston-Salem, North Carolina, ^3^University of Maryland School of Medicine, Baltimore, Maryland; ^4^Michigan State University, East Lansing, Michigan; ^5^Baylor College of Medicine, Houston, Texas

## Abstract

*Dengue is a vectorborne infectious disease caused by dengue viruses (DENVs), which are predominantly transmitted by *Aedes aegypti* and *Aedes albopictus* mosquitos. Dengue is caused by four closely related viruses (DENV-1–4), and a person can be infected with each serotype for a total of four infections during their lifetime. Areas where dengue is endemic in the United States and its territories and freely associated states include Puerto Rico, American Samoa, the U.S. Virgin Islands, the Federated States of Micronesia, the Republic of Marshall Islands, and the Republic of Palau. This report summarizes the recommendations of the Advisory Committee on Immunization Practices (ACIP) for use of the Dengvaxia vaccine in the United States. The vaccine is a live-attenuated, chimeric tetravalent dengue vaccine built on a yellow fever 17D backbone. Dengvaxia is safe and effective in reducing dengue-related hospitalizations and severe dengue among persons who have had dengue infection in the past. Previous natural infection is important because Dengvaxia is associated with an increased risk for severe dengue in those who experience their first natural infection (i.e., primary infection) after vaccination. Dengvaxia was licensed by the Food and Drug Administration for use among children and adolescents aged 9–16 years (referred to in this report as children). ACIP recommends vaccination with Dengvaxia for children aged 9–16 having evidence of a previous dengue infection and living in areas where dengue is endemic. Evidence of previous dengue infection, such as detection of anti-DENV immunoglobulin G with a highly specific serodiagnostic test, will be required for eligible children before vaccination.*

## Introduction

Dengue is a vectorborne infectious disease caused by dengue viruses (DENVs), which are predominantly transmitted by *Aedes aegypti* and *Aedes albopictus* mosquitos. DENVs are members of the genus flavivirus in the family flaviviridae. The four dengue virus serotypes (DENV-1, DENV-2, DENV-3, and DENV-4) all circulate globally, and most countries where dengue is endemic have reported circulation of all four serotypes. These serotypes share structural antigens yet are serologically and genetically distinct.

Dengue is a growing public health challenge (*1*,*2*). Dengue is endemic throughout the tropics and subtropics, with an estimated 3.8 billion persons (95% confidence interval [CI]: 3.5 billion–4.1 billion), or approximately 53% of the global population, living in areas suitable for DENV transmission (*3*). The majority of these areas are in Asia, Africa, and the Americas (*3*). In 2013, an estimated 58 million symptomatic DENV infections (95% CI: 24 million–122 million) and 13,586 deaths occurred worldwide (95% CI: 4,200–34,700), resulting in a total annual cost of $8.9 billion (95% CI: $3.7 billion–$19.7 billion) in direct medical and nonmedical costs and indirect costs associated with time lost because of illness, care, or death (*1*,*2*).

### Pathogenesis

Dengue can be caused by any one of the four distinct but related viruses, and a person can be infected with each serotype for a total of four dengue infections during their lifetime (*4*). After an infection with one DENV serotype, antibodies induced are type specific and cross-react with other DENV serotypes (*4*). The adaptive immune response that develops with infection by any DENV provides long-term immunity to the homologous virus and short-lived protection against heterologous DENV. Human experimental infection studies indicated that this cross-protection lasts approximately 3 months (*5*,*6*), whereas epidemiologic observations suggest that cross-protection might last up to 2 years (*7*,*8*). The risk for severe dengue varies on the basis of many factors, including the number of previous dengue infections a person has had. Whereas any dengue infection can lead to severe dengue, a second infection with a dengue virus is the most likely to cause severe dengue compared with the first and post-secondary infections (*9*,*10*). Multiple mechanisms likely contribute to increased disease severity during a second DENV infection. Cross-reactive or non-neutralizing antibodies binding to a heterologous DENV facilitates uptake in Fc receptor–bearing monocytes and results in both higher magnitude and prolonged viremia (i.e., antibody-dependent enhancement). Moreover, virus-host interactions during antibody-dependent enhancement enable the virus to evade host antiviral and immune responses that would otherwise limit infection (*11*).

An accompanying enhanced immune response also occurs in which activated natural killer cells and memory T-cells trigger inflammatory mediators that contribute to intravascular leakage (*12*). The dengue nonstructural protein 1 (NS1) is secreted from infected cells and is independently associated with vascular leakage by damaging the endothelial glycocalyx and disrupting endothelial cell junctions. This phenomenon might be worsened during a second infection in association with increased viremia (*13*). Although this risk for severe dengue is highest for a second infection with a different DENV serotype, it can occur after post-secondary infection. Previous infection with Zika virus (another flavivirus commonly co-circulating in areas where dengue is endemic) has been demonstrated to increase the risk for symptomatic and severe dengue for subsequent DENV-2 infections occurring several years after Zika infection (*14*). Interactions between dengue and other flaviviruses are less clear (*15*,*16*).

### Dengue Clinical Disease

Dengue clinical disease ranges from a mild, undifferentiated febrile illness to severe disease complicated by shock, bleeding, or severe organ impairment. Approximately 75% of dengue infections are mild or asymptomatic (*17*). The most common presentation of symptomatic disease is sudden onset of fever accompanied by headache, retro-orbital pain, generalized myalgia and arthralgia, flushing of the face, anorexia, abdominal pain, and nausea. A generalized erythematous, macular rash developing within 3–4 days of fever onset frequently is observed. Laboratory-detected findings can include leukopenia, hemoconcentration, transaminitis, and thrombocytopenia. The World Health Organization (WHO) classifies dengue illness as 1) dengue with or without warning signs for progression toward severe dengue and 2) severe dengue (*18*). Warning signs of severe dengue include abdominal pain or tenderness, persistent vomiting, clinical fluid accumulation (e.g., ascites, pericardial effusion, and pleural effusion), mucosal bleeding, lethargy or restlessness, postural hypotension, liver enlargement of >2 cm, or an increased hematocrit level concurrent with a rapid decrease in platelet count (*18*). Criteria for the case definition of severe dengue include any sign of severe plasma leakage leading to shock or fluid accumulation with respiratory distress, severe bleeding, or severe organ impairment.

Patients with severe dengue need in-hospital medical treatment to mitigate poor clinical outcomes commonly due to vascular permeability, which results in plasma leakage and leads to hypovolemic shock or clinically significant ascites or pleural effusions, and less commonly, to severe bleeding due to various host or viral factors (*19*). Because of the risk for complications due to plasma leakage and bleeding, severe dengue requires monitoring and treatment in intensive care settings. Although rare, dengue can affect the liver, heart, central nervous system, kidneys, eyes, muscles, or bone marrow (*4*,*20*,*21*). These severe manifestations of dengue carry a high risk for death and must be recognized and appropriately managed in a timely manner. Age, comorbidities, host genetics, and the infecting virus strain are risk factors for severe dengue, and heterotypic secondary infections are the most prominent factor associated with severe dengue (*4*).

### Dengue Treatment

No effective antiviral treatments against dengue are available; therefore, the mainstay for preventing severe disease and death is timely and supportive management with volume replacement, particularly among patients with severe dengue. The case-fatality ratio for severe dengue has been reported to be as high as 13% (*22*,*23*) and can be <1% with access to timely diagnosis and appropriate treatment (*24*,*25*).

### Dengue Immune Response and Diagnostics

Typically, immunoglobulin M (IgM) antibodies directed against DENV develop during the first week of illness (*26*) and persist for several months to as long as 1 year (*27*). Neutralizing antibodies develop shortly after IgM antibodies and consist primarily of immunoglobulin G (IgG) antibodies. Type-specific neutralizing antibodies persist for many years after dengue and other flavivirus infections (e.g., Zika) and usually confer lifelong immunity to the infecting virus serotype (*28*). In persons previously infected with or vaccinated against a flavivirus, subsequent infection with another flavivirus (i.e., second flavivirus infection) can cause both a diminished IgM response and a rapid increase to high titers of neutralizing antibodies against multiple different flaviviruses, which might prevent conclusive determination of which virus was responsible for the person’s recent infection using serological methods (*29*).

Acute dengue diagnosis can be achieved using blood or serum collected ≤7 days after symptom onset by detection of viral RNA through nucleic acid amplification tests, by detection of viral antigens such as dengue NS1 by enzyme-linked immunosorbent assay (ELISA) or rapid diagnostic tests, and by detection of IgM antibodies through serologic testing. Dengue IgM antibodies start to increase from day 4, with levels peaking between days 10–14 and then declining. In primary dengue infections (i.e., first infection), anti-dengue IgG can be detected at low concentrations by the end of the first week of illness; the antibody concentration increases slowly thereafter and is thought to persist for life. In patients with a previous dengue infection (i.e., had dengue at least once before), anti-dengue IgG titers rise rapidly within the first week of illness (*30*).

Cross-reactivity with Zika virus is reported for all serological assays. Plaque reduction neutralization tests (PRNTs) are quantitative assays that can measure virus-specific neutralizing antibody titers for dengue, Zika, and other flaviviruses to which the patient might have been exposed. For diagnostic testing, CDC uses a PRNT with 90% reduction in the input virus (PRNT90) with a cutoff value titer of ≥10 in serum to define positive specimens (*30*). PRNTs can resolve false-positive IgM antibody results caused by nonspecific reactivity in primary infections and, in certain cases, can help identify the infecting virus, particularly in specimens collected ≥3 months after illness onset. However, in many dengue secondary infections, patients have neutralizing antibody titers that do not allow previous DENV and Zika virus infections to be distinguished (*30*).

### Dengue Prevention

*Ae. aegypti*, the main vector of dengue, has proven difficult to control and continues to expand its geographic range. Control of *Ae. aegypti* is complicated by cryptic and inaccessible breeding sites that make it difficult to locate and control a large proportion of the targeted mosquito population (*31*,*32*). Furthermore, insecticide resistance to *Ae. aegypti* is widespread (*33*,*34*). New regulatory requirements have resulted in discontinuation of some insecticides and greater difficulty in registering new chemicals. *Ae. aegypti* is resistant to all commonly used insecticides in Puerto Rico (*35*,*36*). Successful broad-scale application of integrated vector control management strategies have been difficult to achieve and sustain. The dichlorodiphenyltrichloroethane (DDT) spraying campaign in the 1950s and 1960s across Central and South America nearly eradicated *Ae. aegypti* from the region (*37*), resulting in substantial reductions in disease caused by DENV and yellow fever virus (*38*,*39*). Cuba experienced a substantial reemergence of dengue, leading to a concerted vector control effort that included community mobilization and source reduction and resulted in reductions in the per capita risk for dengue (*40*). However, because of the high cost, such achievements are rare, and their impact in controlling mosquito populations is transient.

### Dengue in the U.S. Territories and Freely Associated States

Areas where dengue is endemic in the United States and its territories and freely associated states include Puerto Rico, American Samoa, the U.S. Virgin Islands, the Federated States of Micronesia, the Republic of Marshall Islands, and the Republic of Palau (*41*). Areas where dengue is endemic are defined as areas with frequent or continuous dengue transmission, with evidence of >10 dengue cases in at least three of the previous 10 years. Dengue epidemics occur in a cyclical pattern every 3–7 years, with all four DENV serotypes reported in the Pacific Islands and in the Caribbean. Of areas where dengue is endemic, Puerto Rico, the U.S. Virgin Islands, and American Samoa report dengue cases to ArboNET (Table 1). Limited surveillance data are available from the Federated States of Micronesia, the Republic of Marshall Islands, and the Republic of Palau.

**TABLE 1:** Reported dengue cases in selected U.S. territories where dengue is endemic — ArboNET, 2010–2020

Year	Puerto Rico		U.S. Virgin Islands		American Samoa	
	No. of cases	Rate*	No. of cases	Rate*	No. of cases	Rate*
2010	10,911	2.9	0	0.0	0	0.0
2011	1,541	0.4	0	0.0	0	0.0
2012	6,025	1.6	142	1.3	0	0.0
2013	9,710	2.6	174	1.6	0	0.0
2014	527	0.1	18	0.2	0	0.0
2015	58	0.0	3	<0.1	0	0.0
2016	204	0.1	11	0.1	1	0.0
2017	11	0.0	1	0.0	508	9.2
2018	3	0.0	0	0.0	150	2.7
2019	76	0.0	3	0.0	0	0.0
2020	772	0.2	0	0.0	0	0.0
**Total**	**29,838**	**0.8**	**352**	**0.3**	**659**	**1.1**

* Per 1,000 population, per year. Rates were calculated using 2010 Census data (https://www.census.gov/data/tables/time-series/demo/popest/2010s-detail-puerto-rico.html, https://www.census.gov/data/datasets/2010/dec/virgin-islands.html; and https://www.census.gov/data/datasets/2010/dec/american-samoa.html).

Approximately 90% of the population at risk for dengue in the U.S. territories and freely associated states live in Puerto Rico. During 2010–2020, approximately 95% of locally acquired dengue cases in the United States (n = 31,289) occurred in Puerto Rico (n = 29,779). During the same period, the greatest number of cases and hospitalizations in Puerto Rico occurred among persons aged 10–19 years, with approximately 11,000 reported cases and 4,000 hospitalizations. Incidence rates also are highest among this age group, ranging from 1 to 7 per 1,000 persons during the most recent outbreak years (2010–2013) based on 2010 census data (https://www.cdc.gov/dengue/statistics-maps/2020.html and https://www.census.gov/data/tables/time-series/demo/popest/2010s-detail-puerto-rico.html). In contrast, during 2010–2020 most dengue deaths in Puerto Rico (88%; 61 of 69) occurred among persons aged 20–89 years (CDC, unpublished data, 2020).

Similar to Puerto Rico, during 2010–2020, persons aged 10–19 years experienced the highest dengue incidence and accounted for the largest number of dengue cases in the U.S. Virgin Islands and American Samoa (https://www.cdc.gov/dengue/statistics-maps/2020.html). Dengue outbreaks were reported from the Federated States of Micronesia in 2011, 2012–2013, 2016, and 2019–2020. In the last outbreak, most cases occurred among persons aged 5–19 years (*42*). Outbreaks also were reported from the Republic of Marshall Islands during 2019–2020 and the Republic of Palau in 2019. Guam and the Northern Mariana Islands have reported sporadic and travel-associated (imported) dengue cases but do not meet the criteria for areas where dengue is endemic (*43*). Hawaii, Texas, Florida, and other states have reported sporadic, locally acquired cases and occasional outbreaks but do not meet the definition of endemic areas (*43*,*44*). During 2010–2017, Hawaii reported 250 locally acquired dengue cases, Florida 103, and Texas 24 (*44*).

Population-based dengue seroprevalence data are not available from any of the U.S. areas where dengue is endemic. However, small convenience studies estimated dengue seroprevalence in Puerto Rico to range from 50% in 2007 (*45*) to 56% in 2010 among participants aged 9–16 years in vaccine trials (*46*). Preliminary results from a community-based study in 2018 in southern Puerto Rico suggested similar seroprevalence in this age group (CDC unpublished data, 2021).

### Dengue Vaccines

Dengvaxia is a live-attenuated, chimeric tetravalent dengue vaccine built on a yellow fever 17D backbone. WHO recommends Dengvaxia for persons aged 9–45 years with confirmed previous DENV infection (*47*). Dengvaxia is licensed in 20 countries. The recommendation is only for persons with confirmed previous DENV infection because the vaccine manufacturer, Sanofi Pasteur, announced that persons not previously infected with DENV who receive Dengvaxia might be at risk for developing severe dengue if they are infected with DENV after being vaccinated (*48*). In May 2019, Dengvaxia was approved by the Food and Drug Administration (FDA) for use in children and adolescents aged 9–16 years (referred to in this report as children) living in an area where dengue is endemic and having had laboratory-confirmed previous DENV infection. Multiple dengue vaccine candidates are in clinical development. Two live-attenuated, tetravalent vaccine candidates are under evaluation in phase 3 trials (*49*,*50*).

Before Dengvaxia received FDA approval, the Advisory Committee on Immunization Practices (ACIP) had no recommendations for the use of vaccines to prevent dengue. This report provides ACIP recommendations for use of Dengvaxia for children aged 9–16 living in areas where dengue is endemic and having evidence of a previous DENV infection. These recommendations are intended to guide public health practitioners and laboratorians in designing and testing vaccination strategies in jurisdictions where DENV transmission is endemic.

## Methods

The Dengue Vaccines Work Group met bimonthly from October 2018 through April 2021 to review Dengvaxia data from clinical trials. The work group comprised ACIP members, including the chair; the CDC lead from the Division of Vector-Borne Disease’s Dengue Branch; experts in dengue and flavivirus epidemiology and vaccinology; representatives of the American Academy of Pediatrics and the Association of Immunization Managers; ex-officio representatives from the FDA, the U.S. Department of Defense, and the National Institute of Allergy and Infectious Diseases; and CDC observers from the National Center for Immunization and Respiratory Diseases, the Division of Global Migration and Quarantine, the Division of Healthcare Quality Promotion, and the Division of Vector-Borne Diseases.

Using the Grading of Recommendations Assessment, Development and Evaluation (GRADE) approach (*51*), the work group defined the research question (i.e., the patient, intervention, comparator, and outcome question), identified critical patient-centered outcomes, systematically reviewed the evidence, assessed the certainty of the evidence, and developed policy options for ACIP’s consideration. The work group identified prevention of the following critical outcomes as benefits: development of virologically confirmed dengue (VCD) (e.g., using a reverse transcription–polymerase chain reaction [RT-PCR] test), severe dengue, and dengue hospitalizations. Outcomes that were considered critical for harms included serious adverse events, hospitalization, severe dengue, and death.

To develop a recommendation using the Evidence to Recommendations Framework (EtR), the work group, assisted by technical experts, reviewed dengue epidemiology, immunology, and pathogenesis; clinical manifestations and management; laboratory diagnostics, including prevaccination screening issues associated with dengue anti-IgG antibody testing; cost-effectiveness; vaccine programmatic implementation and acceptability in Puerto Rico; and health equity issues. Details on the systematic review search and inclusion criteria, summaries of the evidence, and GRADE evidence profiles and the EtR framework are available at https://wwwdev.cdc.gov/vaccines/acip/recs/grade/CYD-TDV-dengue-vaccine-etr.html and https://wwwdev.cdc.gov/vaccines/acip/recs/grade/CYD-TDV-dengue-vaccine.html. ACIP voting members approved vaccination recommendations for children aged 9–16 years with laboratory-confirmed previous DENV infection living in areas of the United States where dengue is endemic.

## Summary of Findings

### Background

Dengvaxia is a prophylactic, tetravalent, live-attenuated, chimeric viral vaccine built on a yellow fever 17D backbone (*52*,*53*). The vaccine includes precursor membrane and envelope genes obtained from each of the four DENV serotypes. Dengvaxia contains four genetic constructs, one for each serotype. The phase 3 randomized, observer-blind, placebo-controlled studies that evaluated efficacy were CYD14 and CYD15. CYD14 included children aged 2–11 years from 11 study sites across the Asia Pacific region, with a total sample size of 10,275 randomized 2:1 to Dengvaxia and placebo. Approximately 2,000 participants who received the vaccine had serostatus determined at baseline. CYD15 included children aged 9–16 years from 22 study sites across Latin America, for a total sample size of 20,869. Approximately 2,000 participants who received the vaccine had serostatus determined at baseline. Three doses of the Dengvaxia vaccine were administered at 0 months, 6 months, and 12 months. Vaccine efficacy against VCD was assessed up to 25 months after vaccination, when the active phase of the trial ended. The studies included continued monitoring of the risk for hospitalization and severe dengue for up to 6 years after the first dose of vaccine during the hospitalization phase (*52*,*53*).

Efficacy results from the phase 3 trials demonstrated excess hospitalizations for dengue among vaccine recipients aged 2–5 years. Efficacy by dengue serostatus could not be ascertained because of the limited sample size with serostatus at baseline. Sanofi Pasteur developed a NS1 IgG ELISA and used subjects’ samples obtained from month 13 to infer their serostatus in a post hoc analysis of safety and efficacy. All cases of VCD, hospitalization, and severe dengue were included, and 10% of participants were randomly selected after stratifying by age and site. The supplemental study used different analytical methods, including multiple imputation (MI), targeted minimum loss-based estimator (TMLE), and the NS1 antibody test results from month 13. Vaccine efficacy was assessed from month 0 onward using MI and TMLE and from month 13 onward using NS1 test results. Results are presented based on the MI results, and efficacy was similar with the NS1 and TMLE approaches (*48*).

### Vaccine Efficacy

Vaccine efficacy against VCD in the per protocol analyses evaluated incidence after completion of the 3-dose schedule and up to 25 months of follow-up. Among seropositive participants aged 9–16 years (n = 1,560), pooled vaccine efficacy against VCD from CYD14 and CYD15 in the immunogenicity subset was 82% (95% CI: 67%–90%), with incidence in unvaccinated and vaccinated seropositive participants of 5% and 1%, respectively (*53*). Using MI, from month 0 to 25 months of follow-up, vaccine efficacy against VCD was 76% (95% CI: 64%–84%) (*48*). Efficacy was highest for DENV-4 (89%; 95% CI: 80%–94%) and lowest for DENV-1 and DENV-2 (67% for each; 95% CI: 46%–80%) (*53*). Among seropositive participants aged 9–16 years, the vaccine was protective against hospitalization (79%; 95% CI: 69%–86%) and severe dengue (84%; 95% CI: 63%–93%) over the 5-year follow-up period. The 5-year incidence of hospitalization for VCD among unvaccinated and vaccinated seropositive participants was 2% and 0.4%, respectively, and for severe dengue was 0.5% and 0.1%, respectively (*48*). Vaccine efficacy against hospitalization decreased from 91% (95% CI: 80%–96%) during the first and second years combined after vaccination to 56% (95% CI: 25%–78%) during the fifth and sixth years combined after vaccination. Vaccine efficacy against hospitalization remained significant throughout the 6-year follow-up period (*54*).

Multiple analyses and trials have explored simplified 1-dose or 2-dose schedules. An analysis of interdose efficacy between doses 1 and 2, doses 2 and 3, and from dose 3 in seropositive participants aged 9–16 years found efficacy of 81% (95% CI: 66%–89%) between doses 1 and 2, 82% (95% CI: 71%–89%) after dose 2, and 74% (95% CI: 66%–82%) after dose 3. These data suggest similar protection against disease even after a single dose (*55*). Antibody responses at 28 days and 1 year in a 1-dose or 2-dose regimen were noninferior compared with a 3-dose regimen against all serotypes in seropositive persons aged 9–50 years (*56*). These data might lead to a recommendation of a simplified dose schedule in the future.

### Vaccine Safety

#### Adverse Reactions

The most frequently reported adverse reactions (n = 1,333), regardless of the dengue serostatus before vaccination, were headache (40%), injection site pain (32%), malaise (25%), asthenia (25%), and myalgia (29%) (*52*). Unsolicited nonserious adverse reactions occurred in 1% of vaccinated participants (16 of 1,333) and in 0.8% of controls (five of 664). No differences were reported in serious adverse events (https://clinicaltrials.gov/ct2/help/adverse_events_desc) at 28 days (0.6% in vaccinated participants and in 0.8% in controls) and deaths in either the vaccine or control arms (0.3% in each group). At 6 months, fewer severe adverse events were reported in the vaccine (3%) than in the control arm (3%). Additional information is available in the package insert (*52*). No dengue-related deaths occurred among participants in the trial.

#### Yellow Fever Backbone

Viscerotropic and neurotropic diseases are rare serious complications associated with yellow fever vaccination (*57*). Although Dengvaxia contains a yellow fever backbone, no cases of viscerotropic or neurotropic illness have been observed (*58*). Vaccine-induced YF-17D-NS3–specific CD8/IFNγ responses have been demonstrated after vaccination with Dengvaxia (*59*); however, no immune response against the structural antigens related to protection against yellow fever has been documented in these vaccine recipients.

#### Vaccinating Seronegative Children

Dengvaxia increases the risk for severe dengue in those who experience their first natural infection after vaccination (*48*). The most important adverse event is hospitalization or severe dengue after vaccinating a seronegative person misclassified as seropositive. Among seronegative children aged 9–16 years over 5 years of follow-up, an overall excess risk for dengue-related hospitalization (relative risk [RR]: 1.41; 95% CI: 0.74–2.68) and severe dengue (RR: 2.44; 95% CI: 0.47–12.56) was reported; however, this excess risk was not statistically significant, likely because of the small sample size (*48*). The possible increased risk for hospitalization and severe dengue among seronegative children is most likely attributable to the vaccine acting as a silent primary DENV infection, thus increasing the risk for severe disease with subsequent natural infection with a DENV (*60*). The cumulative incidence of dengue hospitalization over 5 years among seronegative vaccine recipients was 2% and among placebo recipients was 1%. When stratified by year, the risk for hospitalization among vaccinated seronegative participants compared with seropositive participants was lowest during the first 2 years after vaccination (hazard ratio [HR]: 0.77; 95% CI: 0.26–2.73), peaked during the third year (HR: 2.64; 95% CI: 0.64–10.93), and then progressively declined during the fourth year (HR: 1.68; 95% CI: 0.58–4.89) and the fifth and sixth years (HR: 0.80; 95% CI: 0.48–2.61) (Sanofi Pasteur, personal communication, March 15, 2021).

Among participants aged 9–16 years, clinical signs and symptoms were equivalent in hospitalized vaccinated seronegative participants (n = 56) (hypothesized as a result of vaccine-induced silent primary infection followed by wildtype infection) and hospitalized control seropositive participants (n = 110) (wildtype primary infection followed by wildtype infection). No significant differences were reported in complications between hospitalized vaccinated seronegative participants compared with hospitalized seropositive controls, including hemorrhage (39% versus 42%; RR: 0.94; 95% CI: 0.54–1.59), plasma leakage (36% versus 42%; RR: 0.85; 95% CI: 0.48–1.47), and thrombocytopenia with platelet count ≤50 × 10^9^/L (41% versus 55%; RR: 0.75; 95% CI: 0.44–1.24). The hospitalized vaccinated seronegative participants had no evidence of visceral manifestations or shock, and the seropositive participants in the control group had low rates of each (6%; RR: 0.00; 95% CI: 0.00–1.36 and 2%; RR: 0.00; 95% CI: 0.00–10.36, respectively) (*48*).

### Use in Special Populations

Dengvaxia should be used with precaution in certain populations. Health care providers should weigh the risks of vaccination against the risk for dengue for the following populations. 

#### Pregnant Females

Pregnant females, who are at increased risk for dengue-related complications, were not specifically studied in the Dengvaxia trial. The limited number of pregnant females inadvertently vaccinated during the trial had a similar frequency of adverse pregnancy outcomes (e.g., spontaneous abortion, intrauterine death, and stillbirth) as occurred in the control group; however, the number of vaccinated pregnant females was not sufficient to determine a possible effect of Dengvaxia on pregnancy (*52*).

#### Breastfeeding Females

Human data are not available to evaluate the safety of Dengvaxia on breastfeeding infants. The developmental and health benefits of breastfeeding should be considered in conjunction with the risk for DENV infection in the mother and infant.

#### Persons with HIV Infection

Safety and efficacy of Dengvaxia have not been assessed in persons with HIV infection. However, ongoing studies are assessing the vaccine’s use in adults with well-controlled HIV infection (https://clinicaltrials.gov/ct2/show/NCT02741128).

### Rationale for Recommendations

Dengue is a serious and ongoing public health problem in U.S. territories and freely associated states. Effective and sustainable mosquito control strategies remain elusive, and consistent compliance with personal protective measures is difficult. The intensity of dengue transmission is influenced by population density and ecological factors such as temperature, rainfall, and altitude. The U.S. territories and freely associated states have appropriate conditions for continued dengue and other mosquito-borne diseases transmission.

Dengvaxia is a safe and effective vaccine among persons who have had dengue but is associated with an increased risk for hospitalization and severe dengue among those who have their first natural infection after vaccination. Screening and vaccinating only those who have had a previous laboratory-confirmed infection or who receive positive serologic test results for previous dengue infection offers the potential to retain the benefits of vaccination among seropositive recipients while minimizing the risk for vaccinating seronegative recipients. Screening tests need to be both highly specific to minimize the risk for vaccinating seronegative persons and highly sensitive to ensure a large proportion of seropositive persons are identified and vaccinated. Vaccination should be considered as part of an integrated disease control strategy that includes continuous training for appropriate clinical management and using effective methods for sustained vector control.

## Modeling and Cost-Effectiveness

ACIP reviewed findings from a Dengvaxia cost-effectiveness study (*61*) that based estimates of the costs paid by the government associated with treatment of dengue for ambulatory cases and hospitalizations on estimates from 2002 to 2009 (projected to 2010) in Puerto Rico. Using the Consumer Price Index for medical care for Puerto Rico, the costs were adjusted from 2010 values to 2019 U.S. dollars. The study evaluated the impact of routine vaccination of children aged 9 years in Puerto Rico over 10 years in a scenario of 50% dengue seroprevalence and the implementation of a prevaccination screening laboratory test with 80% sensitivity and 95% specificity. On the basis of direct medical and vaccine program costs, including the screening test, the incremental cost per hospitalization averted was $16,000 and per quality-adjusted life-year (QALY) gained was $122,000 (95% CI: $74,000–$182,000). In a scenario with 30% dengue seroprevalence at age 9 years, the incremental cost per hospitalization averted and QALY gained was $32,000 and $240,000, respectively. The sensitivity analysis indicated that high laboratory screening test specificity is more important than test sensitivity for cost-effectiveness. In addition, test specificity has similar epidemiologic benefit because it would avoid inadvertently vaccinating persons without a previous dengue infection, thereby reducing hospitalizations for severe dengue among this group. The estimates for averted cases and hospitalizations are consistent with other Dengvaxia cost-effectiveness models described in a WHO study (*60*).

### Risk-Benefit Ratio

The model predicts that in a moderate transmission scenario with previous dengue prevalence of 50% in the eligible age group for vaccination, using a serologic screening test with 80% sensitivity and 95% specificity over 10 years (vaccinating children aged 9 years with 80% of children aged 9 years screened), 3,415 hospitalizations would be prevented and an additional 184 hospitalizations would occur; that translates to averting 19 hospitalizations for every additional vaccine-associated hospitalization (*61*). In a lower transmission scenario of 30% prevalence at age 9 years, an estimated 1,162 hospitalizations would be averted and an additional 14 hospitalizations would occur; that translates to averting 8 hospitalizations for every additional vaccine-associated hospitalization. The ratio of hospitalizations averted versus vaccine-induced hospitalizations is improved when using a higher specificity test. Model parameters have been updated with a screening test with 75% sensitivity and 98% specificity. Results indicate that in a 50% seroprevalence scenario, 2,956 hospitalizations would be prevented and an additional 51 hospitalizations would occur; that translates to averting 57 hospitalizations for every additional hospitalization (Guido España, University of Notre Dame, personal communication, April 26, 2021).

### Population Impact

The primary population-level benefit from vaccination will be from the individual level of protection against disease provided to the vaccine recipients. Minimal contribution from indirect effects of decreasing DENV transmission is likely for two reasons. First, approximately 75% of DENV infections are asymptomatic but still induce host viremia that might infect an *Aedes* sp. mosquito vector taking a bloodmeal (*17*). One analysis using pooled data from the phase 3 trials found low vaccine efficacy against asymptomatic disease (34%; 95% CI: 18%–46%) in 2,699 seronegative and seropositive children aged ≥9 years from month 13 to month 25 after the first vaccine dose (*62*). Second, the population eligible for vaccination (aged 9–16 years with a history of DENV infection) composes a relatively small proportion of the entire population at risk for DENV infection, thus requiring many decades of vaccinating successive cohorts to meaningfully increase the level of herd immunity, assuming this low level of efficacy is sustained over time. Multiple studies attempting to model the indirect benefit to unvaccinated persons have demonstrated great variability of the vaccine’s impact on number, timing, and severity of epidemics because of the many factors affecting DENV transmission and uncertainties about long-term vaccine effectiveness (*60*,*63*,*64*).

## Recommendations for the Prevention of Dengue Among the Selected Pediatric Population

ACIP recommends vaccination with the Dengvaxia vaccine for children aged 9–16 years having evidence of a previous dengue infection and living in areas where dengue is endemic. Dengvaxia is recommended as a 3-dose vaccination series, administered 6 months apart at 0, 6, and 12 months, for the selected pediatric population. Evidence of previous dengue infection, such as confirmation with previous laboratory-confirmed infection or a highly specific serodiagnostic test, will be required among eligible children before vaccination.

## Clinical Guidance for Dengue Vaccination Among the Selected Pediatric Population 

### Vaccine Storage and Handling

Store vaccine antigen and saline diluent in a refrigerator at 36°F–46°F (2°C–8°C) and do not freeze. Protect from light. Do not use after the expiration date shown on the vial labels of the lyophilized vaccine antigen and saline diluent. After reconstitution, administer Dengvaxia immediately or store refrigerated at 36°F–46°F (2°C–8°C) and use within 30 minutes. Discard reconstituted vaccine if not used within 30 minutes (*52*).

### Dosage and Administration

Dengvaxia should be administered as 3 doses (0.5 mL each) 6 months apart (at months 0, 6, and 12). After reconstitution, 0.5 mL of Dengvaxia should be immediately administered subcutaneously or stored refrigerated at 36°F–46°F (2°C–8°C) and used within 30 minutes. Dengvaxia is for subcutaneous use only. Dengvaxia should not be administered by intramuscular injection. Additional information is available in the package insert (*52*).

### Vaccine Availability

Dengvaxia is for use only in the U.S. territories and freely associated states where dengue is endemic. Consistent with the indication and ACIP recommendations, Dengvaxia will not be available for purchase or use in areas where dengue is not endemic, including the continental United States, on the basis of the CDC definition of endemicity. The vaccine can be ordered from Sanofi Pasteur by calling 1-800-822-2463.

### Contraindications

#### Vaccine Component Allergy and Immunocompromised Children

Dengvaxia is contraindicated for children with a history of a severe allergic reaction to any component of the vaccine or a previous dose of this vaccine. A complete list of vaccine components is available in the package insert (*52*). Dengvaxia is a live-attenuated vaccine and is contraindicated in children with severe immunodeficiency or immunosuppression due to underlying disease or therapy, including children with symptomatic HIV infection or CD4+ T-lymphocyte count of <200/mm^3^.

### Clinical Considerations

#### Syncope

Syncope can occur before or after vaccination because of a vasovagal response to needles. Children should be seated or lying down during vaccination. Vaccine providers, particularly when vaccinating adolescents, should consider observing patients (with the patient seated or lying) for 15 minutes after vaccination to decrease the risk for injury should the patient faint. If syncope develops, the patient should be observed until the symptoms resolve (https://www.cdc.gov/vaccines/hcp/acip-recs/general-recs/index.html).

#### Anaphylaxis

Although allergic reactions are a concern of vaccine providers, these reactions are uncommon and anaphylaxis after vaccination is rare, occurring at a rate of approximately one per million doses for many vaccines (https://www.cdc.gov/vaccines/hcp/acip-recs/general-recs/index.html). A plan for managing anaphylaxis should be in place. The best practice to prevent allergic reactions is to identify persons at increased risk by obtaining a history and noting allergies to previous vaccines and vaccine components that might indicate an underlying hypersensitivity. Vaccine providers should be familiar with identifying immediate allergic reactions, including anaphylaxis, and be prepared to treat these events at the time of vaccine administration. Providers also should have a plan to contact emergency medical services immediately if a severe acute vaccine reaction occurs (https://www.cdc.gov/vaccines/hcp/acip-recs/general-recs/index.html).

#### Requirement for Vaccination: Laboratory Evidence of Previous Dengue Infection

Because of the excess risk for hospitalizations and severe dengue in seronegative children, Dengvaxia is restricted to use in those with evidence of previous dengue infection. Vaccine providers must evaluate for evidence of previous DENV infection before vaccination to minimize chance of vaccinating seronegative persons. Evidence of previous DENV infection can be fulfilled by a history of laboratory-confirmed dengue according to the 2015 dengue case definition (*65*) or a positive IgG result using a serologic test with the performance characteristics (see CDC Guidance on Dengue Prevaccination Screening Tests Among the Selected Pediatric Population).

### Administration of Dengue Vaccine with Other Vaccines

Multiple studies assessed vaccine safety and immunogenicity in coadministration with other vaccines. Early trials evaluated concomitant administration of Dengvaxia vaccine in infants and toddlers with a yellow fever vaccine (*66*), a pentavalent combination vaccine (diphtheria, tetanus, acellular pertussis, inactivated polio vaccine, and *Haemophilus influenzae* type b) (*67*), and a measles, mumps, and rubella (MMR) vaccine (*68*). No safety issues or decreased immunogenicity were associated with coadministration of these vaccines. Trials are ongoing to evaluate concomitant and sequential administration of the Dengvaxia vaccine with a human papillomavirus vaccine among children aged 9–13 years in Malaysia (*69*) and among children aged 9–14 years in Mexico (*70*), as well as coadministration with tetanus toxoid, reduced diphtheria toxoid, and acellular pertussis vaccine among persons aged 9–60 years (*71*).

Although coadministration with other live-attenuated vaccines including yellow fever and MMR has been evaluated for safety and immunogenicity (*66*,*68*), minimum intervals between administration of Dengvaxia before or after other live vaccines have not been studied. Doses of injectable, live-attenuated vaccines not administered simultaneously should be separated by at least 4 weeks, in accordance with best practice guidance from ACIP (https://www.cdc.gov/vaccines/pubs/pinkbook/genrec.html).

### Reporting of Adverse Events

As for all licensed vaccines, ongoing postmarketing surveillance for rare, serious, and longer-term adverse events associated with administration of Dengvaxia is important for assessing its safety profile. All clinically significant adverse events should be reported to the Vaccine Adverse Events Reporting System (VAERS) at https://vaers.hhs.gov, even if a causal relation to vaccination is unknown or not certain. Reports to VAERS can be made electronically, either online or by fillable PDF form (https://vaers.hhs.gov/esub/index.jsp), or by telephone (800-822-7967). Health care providers are encouraged to report electronically to promote better timeliness and quality of safety data. Long-term safety of the vaccine will be monitored through surveillance of dengue-associated hospitalizations.

Dengue is a reportable disease in the United States. Providers should report all confirmed and presumptive cases of dengue to their local health departments, who will report them to ArboNET, a national electronic passive surveillance system for arboviruses (https://www.cdc.gov/dengue/statistics-maps/2020.html). Hospitalization and severe dengue are variables included in these reports, which local jurisdictions should use to monitor trends in hospitalizations among vaccinated children. Health care providers should be encouraged to report to ensure data completeness.

## CDC Guidance on Dengue Prevaccination Screening Tests Among the Selected Pediatric Population

A history of laboratory-confirmed dengue infection based on the 2015 definition (*15*) or a highly accurate serodiagnostic screening test to determine previous DENV infection is needed before administration of the FDA-licensed dengue vaccine. Results of a specific test detecting anti-DENV IgG antibodies indicate whether a person previously has had dengue infection, and if positive, is eligible to receive the vaccine. Other ways to establish previous DENV infection include documentation of either a positive dengue RT-PCR test result, a positive NS1 antigen test result, or a positive anti-DENV IgM test result in geographic areas without co-circulation of other flaviviruses (e.g., Zika) (*30*). The guidance presented in this report on optimal test performance for anti-DENV IgG screening for U.S. territories and freely associated states is adapted from the international target product profile developed by the Partnership for Dengue Control and the Global Dengue and *Aedes*-transmitted Diseases Consortium (*72*). Key areas are discussed where modification of the international target product profile is recommended for U.S. territories and freely associated states.

Because no screening test is perfect, the goal of this guidance is to minimize the risk that seronegative persons could be misclassified as seropositive while ensuring that most seropositive children who would benefit from vaccine are correctly identified. An anti-DENV IgG test for prevaccination screening should be optimally sensitive and specific to confirm past DENV infection. Additional strategies, such as sequential testing, could be an alternative when tests with adequate performance are more broadly available, with the goal of increasing specificity. Although testing at the point of care is preferable, testing in a laboratory setting might be feasible. A key modification is the recommendation for a test to have ≥75% sensitivity and ≥98% specificity. The positive predictive value (PPV) of a test quantifies the probability that a positive test result is correct or the probability that the person’s seropositive result is in error (1 – PPV). The PPV should be ≥90%. At a stated sensitivity and specificity, tests are more likely to misclassify seronegative persons in low seroprevalence geographic areas than in high seroprevalence areas (Table 2) (*72*,*73*). For example, in populations with seroprevalence of <30%, jurisdictions should use tests with high specificity (>98%) to ensure <10% (1 – PPV) of persons who have positive test results will be misclassified and erroneously vaccinated (Table 2) (*72*,*73*). Conversely, high sensitivity ensures that most seropositive persons would be correctly identified as positive, particularly in high prevalence areas (Table 1). Jurisdictions with high DENV prevalence (e.g., ≥60%) ideally should select a screening test with a negative predictive value of ≥75% to increase identification of persons who would benefit from the vaccine (*74*).

**TABLE 2:** Test performance for a hypothetical dengue screening test in different seroprevalence scenarios

Prevalence in the eligible population (%)	Sensitivity (%)	Specificity (%)	PPV (%)	NPV (%)
30	60	95	84	85
30	70	95	86	88
30	75	95	87	90
30	80	95	87	92
30	90	95	89	96
30	60	98	93	85
30	70	98	94	88
30	75	98	94	90
30	80	98	94	92
30	90	98	95	96
50	60	95	92	70
50	70	95	93	76
50	75	95	94	79
50	80	95	94	83
50	90	95	95	90
50	60	98	97	71
50	70	98	97	77
50	75	98	97	80
50	80	98	98	83
50	90	98	98	91
60	60	95	95	61
60	70	95	95	68
60	75	95	95	72
60	80	95	96	76
60	90	95	96	86
60	60	98	98	62
60	70	98	98	69
60	75	98	98	72
60	80	98	98	77
60	90	98	99	87

**Source:** Adapted with permission from Fongwen N, Wilder-Smith A, Gubler DJ, et al. Target product profile for a dengue pre-vaccination screening test. PLoS Negl Trop Dis 2021;15:e0009557.

**Abbreviations:** NPV = negative predictive value; PPV = positive predictive value.

CDC recommends that an evaluation of new and existing tests for prevaccination screening should be done with a well-characterized panel that includes remote DENV infections (i.e., 1–3 years after documented exposure) of all four DENV serotypes, Zika, and epidemiologically relevant flavivirus infections found in the geographic area where the vaccine is proposed to be used. This evaluation should include samples from persons who received flavivirus vaccines, as well as negative control samples, and should follow international standards (*72*). The evaluation panel should be assessed with 50% and 90% plaque reduction neutralization tests (PRNT50 and PRNT90) to establish test performance in detecting remote DENV monotypic infections. A field evaluation of test performance in an area with previous DENV and Zika or other relevant flavivirus transmission might further help determine sensitivity and specificity.

### Implementation Considerations

Implementation of Dengvaxia vaccination among the selected pediatric population includes considerations such as availability of vaccine; coordination and payment for prevaccination screening; timely and accurate test result interpretation; and implementation, completion, and payment for the 3-dose vaccine schedule. Documentation of previous DENV infection can be used when available, and identification of DENV cases previously reported to public health authorities also might be possible in certain settings. However, because of the large proportion of asymptomatic DENV infections and challenges in obtaining laboratory confirmation of previous DENV test results, serodiagnostic screening to identify evidence of a previous DENV infection will be necessary before vaccination for most eligible children. A prevaccination serodiagnostic screening test for previous DENV infection ideally would be a point-of-care rapid test, enabling vaccination during the same visit as testing.

Jurisdictions will need to evaluate how best to facilitate access to prevaccination screening and results according to local regulations. For example, in Puerto Rico, diagnostic laboratory tests must be performed by a medical technologist appropriately licensed in the jurisdiction according to the Puerto Rico Department of Health Rule 120 (http://ptnet.salud.gov.pr/PTNet%20Documents/Departamento%20de%20Salud-Reglamento%20N%C3%BAm.%20120.pdf). Such a regulatory requirement could lead to a complex, multistep process for parents to obtain a prevaccination screening test order, visit a clinical laboratory for testing, obtain test results, schedule a separate visit with the health care provider to discuss results, and then begin the vaccine series if indicated, often at another location. Local officials will need to identify ways to simplify this process and remove barriers to vaccination, such as offering on-site prevaccination screening at vaccination clinics. Reminders and communication to parents to complete the 3-dose schedule over a 1-year period will be important to maximize protection of children who begin the vaccine series. Educational messages should communicate that Dengvaxia has a vaccine efficacy of 80%, and certain persons can have breakthrough dengue infections after complete vaccination. Most hospitalizations among children who have been vaccinated will be breakthrough infections. A pilot project or phased implementation to identify and mitigate potential logistical issues concerning the requirement for prevaccination screening of children in a setting such as Puerto Rico would be desirable.

The Vaccines for Children (VFC) program helps provide vaccines to children whose parents or guardians might not be able to afford them. Persons are eligible for the VFC program if they are aged <19 years and Medicaid eligible, uninsured, underinsured, or an American Indian/Alaska Native. The costs associated with vaccination are paid by private insurers without cost-sharing as mandated for vaccinations recommended by ACIP in Section 2713 of the Public Health Service Act, as added by the Affordable Care Act and incorporated into the Employee Retirement Income Security Act of 1974 (*75*). Cost of the screening test will be covered by Medicaid for those who are eligible and by insurance companies for those who are insured.

### Community Acceptance

A parent survey conducted in southern Puerto Rico in 2018 (n = 1,139) indicated that 75% of parents were interested in having their children vaccinated against dengue (*76*). Of those who were uncertain or not interested, vaccine side effects were the most frequently mentioned concern. In focus groups, most parents said they would agree to have their children vaccinated with Dengvaxia after they received sufficient information about the vaccine. Pediatricians surveyed in Puerto Rico during 2019–2020 (n = 115) considered dengue to be a significant public health problem (*76*). Most pediatricians surveyed (73%) responded they would recommend use of the vaccine if a laboratory test with acceptable specificity was available to document previous DENV infection. Pediatricians who responded that they were unsure about or would not vaccinate with Dengvaxia were concerned about the risks for inadvertently vaccinating persons with a false-positive DENV laboratory test result. Key stakeholders interviewed, including pediatricians, school principals, and school nurses, were all receptive to the Dengvaxia vaccination program for children with laboratory-confirmed evidence of previous DENV infection.

In the U.S. Virgin Islands, a survey of 11 clinicians representing 11 health care facilities in November 2020 found that 64% of facilities were not aware that there was an FDA-approved vaccine (CDC Dengue Branch, unpublished data, 2020). Assuming a laboratory test with acceptable specificity was available, 46% reported they would recommend the vaccine, and 63% responded that they would need more information. Four clinicians (36%) reported that dengue is not an important enough health problem in the U.S. Virgin Islands to justify the cost of a vaccination program. Data on vaccine acceptability are not available for other areas of the United States where dengue is endemic and would be important to obtain before implementing a vaccination program.

Providing communication materials to vaccine providers, parents, and the public to describe the screening test and vaccination plan for administering Dengvaxia will be critical. Culturally appropriate messaging strategies tailored to local jurisdictions’ vaccination programs should be developed before vaccine rollout to ensure high levels of community support.

Local jurisdictions should prepare materials before dengue epidemics to clearly communicate to parents and the public the low risk for inadvertently vaccinating seronegative children with use of highly accurate tests. Because Dengvaxia vaccine efficacy is approximately 80% (*48*), messaging should communicate that although Dengvaxia reduces overall hospitalizations and severe dengue in vaccine recipients, breakthrough DENV infections and hospitalizations will occur in approximately 20% of vaccinated children. Therefore, all patients with symptoms consistent with dengue should be evaluated and treated according to established guidelines, regardless of vaccination status.

## Future Research

Research on optimal use of Dengvaxia and other dengue vaccines likely available in the future is needed. Additional data on seroprevalence in areas of the United States where dengue is endemic and in travelers, including subgroups such as foreign-born persons from regions where dengue is endemic, would enable more precise assessment of geographic areas and groups that could benefit from dengue vaccination. Development of both highly specific and highly sensitive laboratory tests, including algorithms with confirmatory testing, are needed to minimize the chance of vaccinating persons without a previous DENV infection and to maximize benefit to persons previously infected with DENV (*77*). Behavioral science research should guide the development of communication materials that clearly and transparently explain the dengue vaccine’s risks and benefits to the public in English, Spanish, and other languages commonly spoken in the United States and U.S. territories (*76*,*78*). Operational research can assist with the design of efficient dengue vaccination programs that involve prevaccination laboratory screening tests and link test results to medical records and vaccination registries. Ideally, screening and vaccination could be completed in one health care visit, and the program would have high rates of timely vaccine series completion. Vaccine effectiveness studies will be important to monitor vaccine efficacy and vaccination coverage in the context of programmatic dengue vaccine implementation as well as the associated overall population impact in reducing disease. Until a highly efficacious quadrivalent dengue vaccine that provides balanced protection against all four DENV serotypes is available, clinical trials examining the immunogenicity of sequential vaccination with monovalent dengue vaccines or combinations of multivalent vaccines with unbalanced protection by serotype might lead to the identification of alternative vaccination strategies that provide a high level of protection against dengue disease (*79*).

## References

[1] Stanaway JD, Shepard DS, Undurraga EA, The global burden of dengue: an analysis from the Global Burden of Disease Study 2013. Lancet Infect Dis 2016;16:712–23. 10.1016/S1473-3099(16)00026-8 26874619 PMC5012511

[2] Shepard DS, Undurraga EA, Halasa YA, Stanaway JD. The global economic burden of dengue: a systematic analysis. Lancet Infect Dis 2016;16:935–41. 10.1016/S1473-3099(16)00146-8 27091092

[3] Messina JP, Brady OJ, Golding N, The current and future global distribution and population at risk of dengue. Nat Microbiol 2019;4:1508–15. 10.1038/s41564-019-0476-8 31182801 PMC6784886

[4] Wilder-Smith A, Ooi E-E, Horstick O, Wills B. Dengue. Lancet 2019;393:350–63. 10.1016/S0140-6736(18)32560-1 30696575

[5] Snow GE, Haaland B, Ooi EE, Gubler DJ. Review article: research on dengue during World War II revisited. Am J Trop Med Hyg 2014;91:1203–17. 10.4269/ajtmh.14-0132 25311700 PMC4257648

[6] Sabin AB. Research on dengue during World War II. Am J Trop Med Hyg 1952;1:30–50. 10.4269/ajtmh.1952.1.30 14903434

[7] Montoya M, Gresh L, Mercado JC, Symptomatic versus inapparent outcome in repeat dengue virus infections is influenced by the time interval between infections and study year. PLoS Negl Trop Dis 2013;7:e2357. 10.1371/journal.pntd.0002357 23951377 PMC3738476

[8] Anderson KB, Gibbons RV, Cummings DAT, A shorter time interval between first and second dengue infections is associated with protection from clinical illness in a school-based cohort in Thailand. J Infect Dis 2014;209:360–8. 10.1093/infdis/jit436 23964110 PMC3883164

[9] Halstead SB, Nimmannitya S, Cohen SN. Observations related to pathogenesis of dengue hemorrhagic fever. IV. Relation of disease severity to antibody response and virus recovered. Yale J Biol Med 1970;42:311–28. 5419206 PMC2591704

[10] Sangkawibha N, Rojanasuphot S, Ahandrik S, Risk factors in dengue shock syndrome: a prospective epidemiologic study in Rayong, Thailand. I. The 1980 outbreak. Am J Epidemiol 1984;120:653–69. 10.1093/oxfordjournals.aje.a113932 6496446

[11] Ong EZ, Zhang SL, Tan HC, Gan ES, Chan KR, Ooi EE. Dengue virus compartmentalization during antibody-enhanced infection. Sci Rep 2017;7:40923. 10.1038/srep40923 28084461 PMC5234037

[12] Srikiatkhachorn A, Mathew A, Rothman AL. Immune-mediated cytokine storm and its role in severe dengue. Semin Immunopathol 2017;39:563–74. 10.1007/s00281-017-0625-1 28401256 PMC5496927

[13] Glasner DR, Puerta-Guardo H, Beatty PR, Harris E. The good, the bad, and the shocking: the multiple roles of dengue virus nonstructural protein 1 in protection and pathogenesis. Annu Rev Virol 2018;5:227–53. 10.1146/annurev-virology-101416-041848 30044715 PMC6311996

[14] Katzelnick LC, Narvaez C, Arguello S, Zika virus infection enhances future risk of severe dengue disease. Science 2020;369:1123–8. 10.1126/science.abb6143 32855339 PMC8274975

[15] Luppe MJ, Verro AT, Barbosa AS, Nogueira ML, Undurraga EA, da Silva NS. Yellow fever (YF) vaccination does not increase dengue severity: a retrospective study based on 11,448 dengue notifications in a YF and dengue endemic region. Travel Med Infect Dis 2019;30:25–31. 10.1016/j.tmaid.2019.05.002 31075425

[16] Anderson KB, Gibbons RV, Thomas SJ, Preexisting Japanese encephalitis virus neutralizing antibodies and increased symptomatic dengue illness in a school-based cohort in Thailand. PLoS Negl Trop Dis 2011;5:e1311. 10.1371/journal.pntd.0001311 21991398 PMC3186763

[17] Grange L, Simon-Loriere E, Sakuntabhai A, Gresh L, Paul R, Harris E. Epidemiological risk factors associated with high global frequency of inapparent dengue virus infections. Front Immunol 2014;5:280. 10.3389/fimmu.2014.00280 24966859 PMC4052743

[18] World Health Organization and the Special Programme for Research and Training in Tropical Diseases. Dengue: guidelines for diagnosis, treatment, prevention and control. Geneva, Switzerland: World Health Organization; 2009.

[19] Chuang Y-C, Lin Y-S, Liu C-C, Factors contributing to the disturbance of coagulation and fibrinolysis in dengue virus infection. J Formos Med Assoc 2013;112:12–7. 10.1016/j.jfma.2012.10.013 23332424

[20] Lee TH, Lee LK, Lye DC, Leo YS. Current management of severe dengue infection. Expert Rev Anti Infect Ther 2017;15:67–78. 10.1080/14787210.2017.1248405 27786589

[21] Halstead S, Wilder-Smith A. Severe dengue in travellers: pathogenesis, risk and clinical management. J Travel Med 2019;26:taz062. 10.1093/jtm/taz062 31423536

[22] Kalayanarooj S. Standardized clinical management: evidence of reduction of dengue haemorrhagic fever case-fatality rate in Thailand. Dengue Bull 1999;23:10–7.

[23] Kabra SK, Verma IC, Arora NK, Jain Y, Kalra V. Dengue haemorrhagic fever in children in Delhi. Bull World Health Organ 1992;70:105–8. 1568274 PMC2393341

[24] Wills BA, Nguyen MD, Ha TL, Comparison of three fluid solutions for resuscitation in dengue shock syndrome. N Engl J Med 2005;353:877–89. 10.1056/NEJMoa044057 16135832

[25] Lam PK, Tam DT, Diet TV, Clinical characteristics of dengue shock syndrome in Vietnamese children: a 10-year prospective study in a single hospital. Clin Infect Dis 2013;57:1577–86. 10.1093/cid/cit594 24046311 PMC3814826

[26] Martin DA, Muth DA, Brown T, Johnson AJ, Karabatsos N, Roehrig JT. Standardization of immunoglobulin M capture enzyme-linked immunosorbent assays for routine diagnosis of arboviral infections. J Clin Microbiol 2000;38:1823–6. 10.1128/JCM.38.5.1823-1826.2000 10790107 PMC86599

[27] Chien Y-W, Liu Z-H, Tseng F-C, Prolonged persistence of IgM against dengue virus detected by commonly used commercial assays. BMC Infect Dis 2018;18:156. 10.1186/s12879-018-3058-0 29609533 PMC5880084

[28] Poland JD, Calisher CH, Monath TP, Downs WG, Murphy K. Persistence of neutralizing antibody 30–35 years after immunization with 17D yellow fever vaccine. Bull World Health Organ 1981;59:895–900. 6978196 PMC2396120

[29] Halstead SB, Rojanasuphot S, Sangkawibha N. Original antigenic sin in dengue. Am J Trop Med Hyg 1983;32:154–6. 10.4269/ajtmh.1983.32.154 6824120

[30] Sharp TM, Fischer M, Muñoz-Jordán JL, Dengue and Zika virus diagnostic testing for patients with a clinically compatible illness and risk for infection with both viruses. MMWR Recomm Rep 2019;68(No. RR-1). 10.15585/mmwr.rr6801a1 31194720 PMC6581290

[31] Burke R, Barrera R, Lewis M, Kluchinsky T, Claborn D. Septic tanks as larval habitats for the mosquitoes *Aedes aegy*pti and *Culex quinquefasciatus* in Playa-Playita, Puerto Rico. Med Vet Entomol 2010;24:117–23. 10.1111/j.1365-2915.2010.00864.x 20374477

[32] Barrera R, Amador M, Diaz A, Smith J, Munoz-Jordan JL, Rosario Y. Unusual productivity of *Aedes aegypti* in septic tanks and its implications for dengue control. Med Vet Entomol 2008;22:62–9. 10.1111/j.1365-2915.2008.00720.x 18380655

[33] Hemingway J, Boddington R, Harris J, Dunbar S. Mechanisms of insecticide resistance in *Aedes aegypti* (L.) (Diptera: Culicidae) from Puerto Rico. Bull Entomol Res 1989;79:123–30. 10.1017/S0007485300018630

[34] Achee NL, Grieco JP, Vatandoost H, Correction: alternative strategies for mosquito-borne arbovirus control. PLoS Negl Trop Dis 2019;13:e0007275. 10.1371/journal.pntd.0007275 30913223 PMC6435112

[35] Hemme RR, Vizcaino L, Harris AF, Rapid screening of *Aedes aegypti* mosquitoes for susceptibility to insecticides as part of Zika emergency response, Puerto Rico. Emerg Infect Dis 2019;25:1959–61. 10.3201/eid2510.181847 31538919 PMC6759244

[36] Ponce-García G, Del Río-Galvan S, Barrera R, Knockdown resistance mutations in *Aedes aegypti* (Diptera: Culicidae) from Puerto Rico. J Med Entomol 2016;53:1410–4. 10.1093/jme/tjw115 27493252

[37] Schliessmann DJ. *Aedes aegypti* eradication program of the United States—progress report 1965. Am J Public Health Nations Health 1967;57:460–5. 10.2105/AJPH.57.3.460 6066906 PMC1227178

[38] Gubler DJ. Dengue and dengue hemorrhagic fever in the Americas. P R Health Sci J 1987;6:107–11. 3313490

[39] Soper FL. The elimination of urban yellow fever in the Americas through the eradication of *Aedes aegypti.* Am J Public Health Nations Health 1963;53:7–16. 10.2105/AJPH.53.1.7 13978257 PMC1253857

[40] Kourí G, Guzmán MG, Valdés L, Reemergence of dengue in Cuba: a 1997 epidemic in Santiago de Cuba. Emerg Infect Dis 1998;4:89–92. 10.3201/eid0401.980111 9454563 PMC2627664

[41] Jentes ES, Lash RR, Johansson MA, Evidence-based risk assessment and communication: a new global dengue-risk map for travellers and clinicians. J Travel Med 2016;23:taw062. 10.1093/jtm/taw062 27625400 PMC5345513

[42] Government of the Federated States of Micronesia. Dengue virus type-3 outbreak, Yap State: situational report #8 epi week #37: September 9–15, 2019—report date: September 19, 2019. Micronesia (Federated States of) September 19, 2019.

[43] CDC. CDC yellow book 2020: health information for international travel. New York, City NY: Oxford University Press; 2017

[44] Rivera A, Adams LE, Sharp TM, Lehman JA, Waterman SH, Paz-Bailey G. Travel-associated and locally acquired dengue cases—United States, 2010–2017. MMWR Morb Mortal Wkly Rep 2020;69:149–54. 10.15585/mmwr.mm6906a1 32053577 PMC7017959

[45] Argüello DF, Tomashek KM, Quiñones L, Incidence of dengue virus infection in school-aged children in Puerto Rico: a prospective seroepidemiologic study. Am J Trop Med Hyg 2015;92:486–91. 10.4269/ajtmh.14-0231 25646256 PMC4350535

[46] L’Azou M, Assoukpa J, Fanouillere K, Dengue seroprevalence: data from the clinical development of a tetravalent dengue vaccine in 14 countries (2005–2014). Trans R Soc Trop Med Hyg 2018;112:158–68. 10.1093/trstmh/try037 29800279 PMC5972646

[47] Dengue vaccine: WHO position paper, September 2018—recommendations. Vaccine 2019;37:4848–9. 10.1016/j.vaccine.2018.09.063 30424888

[48] Sridhar S, Luedtke A, Langevin E, Effect of dengue serostatus on dengue vaccine safety and efficacy. N Engl J Med 2018;379:327–40. 10.1056/NEJMoa1800820 29897841

[49] Biswal S, Borja-Tabora C, Martinez Vargas L, ; TIDES study group. Efficacy of a tetravalent dengue vaccine in healthy children aged 4–16 years: a randomised, placebo-controlled, phase 3 trial. Lancet 2020;395:1423–33. 10.1016/S0140-6736(20)30414-1 32197105

[50] Whitehead SS. Development of TV003/TV005, a single dose, highly immunogenic live attenuated dengue vaccine; what makes this vaccine different from the Sanofi-Pasteur CYD™ vaccine? Expert Rev Vaccines 2016;15:509–17. 10.1586/14760584.2016.1115727 26559731 PMC4956407

[51] CDC. GRADE (Grading of Recommendations, Assessment, Development and Evaluation). Atlanta, GA: US Department of Health and Human Services, CDC; 2019.

[52] Dengvaxia [Package Insert]. Swiftwater, PA: Sanofi; 2019.

[53] Hadinegoro SR, Arredondo-García JL, Capeding MR, ; CYD-TDV Dengue Vaccine Working Group. Efficacy and long-term safety of a dengue vaccine in regions of endemic disease. N Engl J Med 2015;373:1195–206. 10.1056/NEJMoa1506223 26214039

[54] Forrat R, Dayan GH, DiazGranados CA, Analysis of hospitalized and severe dengue cases over the 6 years of follow-up of the tetravalent dengue vaccine (CYD-TDV) efficacy trials in Asia and Latin America. Clin Infect Dis 2021;73:1003–12. 10.1093/cid/ciab288 33822015 PMC8442794

[55] Dayan GH, Langevin E, Forrat R, Efficacy after 1 and 2 doses of CYD-TDV in dengue endemic areas by dengue serostatus. Vaccine 2020;38:6472–7. 10.1016/j.vaccine.2020.07.056 32773243

[56] Coronel-MartÍnez DL, Park J, López-Medina E, Immunogenicity and safety of simplified vaccination schedules for the CYD-TDV dengue vaccine in healthy individuals aged 9–50 years (CYD65): a randomised, controlled, phase 2, non-inferiority study. Lancet Infect Dis 2021;21:517–28. 10.1016/S1473-3099(20)30767-2 33212067

[57] Staples JE, Gershman M, Fischer M; CDC. Yellow fever vaccine: recommendations of the Advisory Committee on Immunization Practices (ACIP). MMWR Recomm Rep 2010;59(No. RR-7). 20671663

[58] Godói IP, Lemos LLP, de Araújo VE, Bonoto BC, Godman B, Guerra Júnior AA. CYD-TDV dengue vaccine: systematic review and meta-analysis of efficacy, immunogenicity and safety. J Comp Eff Res 2017;6:165–80. 10.2217/cer-2016-0045 28084784

[59] Harenberg A, Begue S, Mamessier A, Persistence of Th1/Tc1 responses one year after tetravalent dengue vaccination in adults and adolescents in Singapore. Hum Vaccin Immunother 2013;9:2317–25. 10.4161/hv.25562 23839107 PMC3981840

[60] Flasche S, Jit M, Rodríguez-Barraquer I, The long-term safety, public health impact, and cost-effectiveness of routine vaccination with a recombinant, live-attenuated dengue vaccine (Dengvaxia): a model comparison study. PLoS Med 2016;13:e1002181. 10.1371/journal.pmed.1002181 27898668 PMC5127514

[61] España G, Leidner AJ, Waterman SH, Perkins TA. Cost-effectiveness of dengue vaccination in Puerto Rico. PLoS Negl Trop Dis 2021;15:e0009606. 10.1371/journal.pntd.0009606 34310614 PMC8341694

[62] Olivera-Botello G, Coudeville L, Fanouillere K, ; CYD-TDV Vaccine Trial Group. Tetravalent dengue vaccine reduces symptomatic and asymptomatic dengue virus infections in healthy children and adolescents aged 2–16 years in Asia and Latin America. J Infect Dis 2016;214:994–1000. 10.1093/infdis/jiw297 27418050 PMC5021228

[63] Ortega-Sanchez IR, Lee GM, Jacobs RJ, ; Working Group on Leading Economic Issues for New Vaccine for Adolescents. Projected cost-effectiveness of new vaccines for adolescents in the United States. Pediatrics 2008;121(Suppl 1):S63–78. 10.1542/peds.2007-1115H 18174323

[64] Perkins TA, Reiner RC Jr, España G, An agent-based model of dengue virus transmission shows how uncertainty about breakthrough infections influences vaccination impact projections. PLOS Comput Biol 2019;15:e1006710. 10.1371/journal.pcbi.1006710 30893294 PMC6443188

[65] CDC. Dengue virus infections 2015 case definition. Atlanta, GA: US Department of Health and Human Services, CDC; 2015.

[66] López P, Lanata CF, Zambrano B, Immunogenicity and safety of yellow fever vaccine (Stamaril) when administered concomitantly with a tetravalent dengue vaccine candidate in healthy toddlers at 12–13 months of age in Colombia and Peru: a randomized trial. Pediatr Infect Dis J 2016;35:1140–7. 10.1097/INF.0000000000001250 27254034

[67] Melo FIR, Morales JJR, De Los Santos AHM, Rivas E, Vigne C, Noriega F. Immunogenicity and safety of a booster injection of DTap-IPV//Hib (Pentaxim) administered concomitantly with tetravalent dengue vaccine in healthy toddlers 15–18 months of age in Mexico: a randomized trial. Pediatr Infect Dis J 2017;36:602–8. 10.1097/INF.0000000000001542 28067718

[68] Crevat D, Brion JD, Gailhardou S, Laot TM, Capeding MR. First experience of concomitant vaccination against dengue and MMR in toddlers. Pediatr Infect Dis J 2015;34:884–92. 10.1097/INF.0000000000000752 25966916

[69] Hassan J, Toh T-H, Sivapunniam SK, Immunogenicity and safety of a tetravalent dengue vaccine administered concomitantly or sequentially with quadrivalent human papillomavirus vaccine in boys and girls 9–13 years of age in Malaysia: a phase IIIb, randomized, open-label study. Pediatr Infect Dis J 2021;40:774–81. 10.1097/INF.0000000000003164 34250977 PMC8274580

[70] Arredondo JL, Villagomez Martinez SM, Concepcion Morales M, Immunogenicity and safety of a tetravalent dengue vaccine and a bivalent HPV vaccine given concomitantly or sequentially in girls aged 9 to 14 years in Mexico. Vaccine 2021;39:3388–96. 10.1016/j.vaccine.2021.04.064 33992441

[71] Santos J, Montellano ME, Solante R, Immunogenicity and safety of a tetravalent dengue vaccine administered concomitantly or sequentially with Tdap vaccine: randomized phase IIIb trial in healthy participants 9–60 years of age in the Philippines. Pediatr Infect Dis J 2021;40:856–63. 10.1097/INF.0000000000003220 34117198 PMC8357045

[72] Fongwen N, Wilder-Smith A, Gubler DJ, Target product profile for a dengue pre-vaccination screening test. PLoS Negl Trop Dis 2021;15:e0009557. 10.1371/journal.pntd.0009557 34324505 PMC8320982

[73] Rodríguez-Barraquer I, Salje H, Cummings DA. Dengue pre-vaccination screening and positive predictive values. Lancet Infect Dis 2019;19:132–4. 10.1016/S1473-3099(18)30799-0 30712834

[74] Flasche S, Smith PG. Sensitivity and negative predictive value for a rapid dengue test. Lancet Infect Dis 2019;19:465–6. 10.1016/S1473-3099(19)30167-7 31034385

[75] CDC. Preventive service tables: prevention through health care. Atlanta, GA: US Department of Health and Human Services, CDC; 2020.

[76] Esquilin I. Dengue vaccine knowledge and attitudes in Puerto Rico. Presented at the Advisory Committee on Immunization Practices Dengue Virus Vaccines meeting, Atlanta, GA; February 27, 2020.

[77] Wilder-Smith A, Peeling RW. Optimising dengue pre-vaccination screening. Lancet Infect Dis 2021;21:442–4. 10.1016/S1473-3099(20)30722-2 33212066 PMC7834846

[78] Hombach J. WHO global position on dengue vaccination. Presented at the Advisory Committee on Immunization Practices Dengue Virus Vaccines meeting, Atlanta, GA; February 27, 2020.

[79] Wilder-Smith A. Dengue vaccine development by the year 2020: challenges and prospects. Curr Opin Virol 2020;43:71–8. 10.1016/j.coviro.2020.09.004 33086187 PMC7568693

